# Bakuchiol, a Natural Antioxidant, Synergizes with Colistin Against Multidrug-Resistant Gram-Negative Bacteria by Disrupting Iron Homeostasis and Membrane Integrity

**DOI:** 10.3390/antiox14101178

**Published:** 2025-09-26

**Authors:** Qianqian Li, Shaobo Yun, Xiaomin Ren, Sijie Wu, Jia Cheng, Xiaoyong Huang

**Affiliations:** College of Veterinary Medicine, Shanxi Agricultural University, Taiyuan 030031, China; liqianqian25@sxau.edu.cn (Q.L.); yuanshaobo1@sxau.edu.cn (S.Y.); renxiaomin@sxau.edu.cn (X.R.); wusijie1@sxau.edu.cn (S.W.)

**Keywords:** bakuchiol, iron, colistin, membrane, synergist, PVP

## Abstract

The rapid emergence and global dissemination of colistin resistance pose a critical threat to public health by limiting therapeutic options against Gram-negative infections. In this study, we report that bakuchiol (BAK), a natural antioxidant meroterpenoid, significantly restores colistin (COL) efficacy against a range of clinically relevant Gram-negative pathogens, including colistin-resistant strains. The combination of BAK and COL reduced the minimum inhibitory concentrations (MICs) of colistin by 8- to 32-fold, indicating strong synergistic antibacterial activity. Mechanistic studies revealed that BAK disrupts bacterial iron homeostasis via chelation-mediated intracellular iron depletion and concurrently compromises membrane integrity through interaction with phospholipids. This dual action induces collapse of the proton motive force and severe metabolic dysfunction. Importantly, the BAK–COL combination exhibited no detectable toxicity and demonstrated potent in vivo efficacy in a *Galleria mellonella* infection model. Furthermore, formulation with polyvinylpyrrolidone (PVP) markedly improved the solubility and sustained the synergistic effects of BAK over a broad concentration range. Our findings highlight the potential of bakuchiol as a novel antioxidant adjuvant and provide a promising combinatory strategy for overcoming multidrug-resistant bacterial infections.

## 1. Introduction

Polymyxins, including colistin (polymyxin E), were historically restricted in clinical use due primarily to nephrotoxicity concerns [1]. However, the relentless rise of extensively antimicrobial-resistant (AMR) Gram-negative bacteria, such as carbapenem-resistant Enterobacteriaceae (CRE) and *Acinetobacter baumannii*, has necessitated the critical reintroduction of this antibiotic class [2,3]. Consequently, polymyxins have regained status as a vital “last-line” defense against these formidable pathogens when other therapeutic options are exhausted. Colistin exerts its bactericidal effect primarily by binding to lipid A, a component of lipopolysaccharide (LPS) in the bacterial outer membrane. This binding disrupts membrane integrity, leading ultimately to cell lysis and death. Unfortunately, this critical reliance on polymyxins has been severely compromised by the emergence and global dissemination of the mobile colistin resistance (*mcr*) genes [4]. Plasmid-borne *mcr* genes encode phosphoethanolamine transferases that modify lipid A, significantly reducing its affinity for polymyxins. The rapid horizontal spread of *mcr* genes across diverse bacterial species, including Enterobacterales, *Klebsiella pneumoniae*, *Acinetobacter baumannii*, and *Pseudomonas aeruginosa*, and geographical regions has alarmingly eroded colistin’s clinical efficacy [5]. Therefore, novel therapeutic strategies are urgently needed to overcome *mcr*-mediated resistance, preserve the utility of existing polymyxins, and combat infections caused by these pan-resistant pathogens.

A numerous of synthetic compounds have demonstrated the ability to enhance the antibacterial activity of colistin against resistant strains by circumventing resistance mechanisms or synergistically targeting bacterial vulnerabilities [6,7,8]. Notably, natural compounds represent a particularly promising and underexplored class of potentiators. Derived from diverse biological sources such as plants, fungi, and microorganisms, these naturally occurring molecules offer a rich reservoir of novel chemical scaffolds with inherent antibacterial or resistance-modifying properties [9,10,11,12]. Natural flavonoids, such as α-mangostin, isobavachalcone [13], glabrol [14], myricetin, or luteolin [15], not only exhibit rapid bactericidal effects against Gram-positive bacteria but also reverse colistin resistance in Gram-negative bacteria by disrupting bacterial iron homeostasis. Bakuchiol (BAK) is a naturally derived prenylated phenolic meroterpenoid, primarily isolated from the seeds of *Psoralea corylifolia* (commonly known as “Bu-gu-zhi” in Chinese) [16]. This le-guminous plant is widely used in traditional Chinese medicine and is predominantly cultivated in several Chinese provinces such as Henan, Yunnan, and Guizhou. The extraction of BAK is commonly performed using organic solvents like ethanol [17]. Due to its promising biological properties, BAK has gained growing interest in scientific research. Traditionally used in Ayurvedic and Chinese medicine, BAK exhibits a broad spectrum of pharmacological properties, including potent antioxidant, anti-inflammatory, and antimicrobial activities [18,19,20,21]. Its antioxidant capacity, in particular, is linked to its ability to modulate cellular redox states, a property that may be exploited to disrupt the delicate physiological balance in bacterial cells. Recently, it has demonstrated that BAK potentiate colistin efficacy against *Acinetobacter baumannii* persisters [22]. However, the underlying molecular mechanism of action remains poorly understood.

Here, we investigated the potent synergistic activity between BAK and COL against diverse AMR Gram-negative pathogens. Mechanistically, this synergy requires BAK-induced metabolic perturbation, increased membrane permeability, and iron homeostasis dysregulation. Critically, the translational relevance of this combination is substantiated through *Galleria mellonella* infection studies, confirming enhanced survival rates in treated larvae without observable toxicity, thereby supporting its therapeutic feasibility.

## 2. Materials and Methods

### 2.1. Bacterial Strains and Chemicals

The following strains were used: *Escherichia coli* (*E. coli*) ATCC25922 (Standard strain), *E. coli* B2 (multidrug-resistant), Uro-pathogenic *E. coli* (UPEC) strains CFT675 and J96, *E. coli* Nissle1917 (type strain), *E. coli* CMCC44102 (Standard strain), *Klebsiella pneumoniae* CAU1595 (carbapenem-resistant), *Pseudomonas aeruginosa* PA14 (wild type), *Salmonella typhimurium* CAU2005 (carbapenem-resistant), *Acinetobacter baumannii (A. baumannii)* ATCC17978 (Standard strain), *A. baumannii* AB34 (carbapenem-resistant), *Salmonella enterica* CAU193 (carbapenem-resistant). The multidrug-resistant *E. coli* B2, carrying 25 antibiotic resistance genes [23], was selected as a representative model for further mechanistic investigation. All strains were cultured in Luria–Bertani (LB) broth (Land Bridge, Beijing, China) at 37 °C with 220 rpm shaking. Unless otherwise indicated, bacterial cultures should be grown to logarithmic phase under shaking conditions prior to use. Bakuchiol (BAK; CAS:10309-37-2, purity ≥ 98%, Aikon, Nanjing, China) was dissolved in DMSO to prepare stock solutions (10 mg/mL or 100 mM). Colistin (COL; Bide, Shanghai, China) was prepared in sterilized distilled water.

### 2.2. Antimicrobial Susceptibility Testing

Minimum inhibitory concentrations (MICs) were determined by the broth microdilution method in cation-adjusted Mueller-Hinton broth (CAMHB, Solarbio, Beijing, China) following CLSI (Clinical and Laboratory Standards Institute) recommendations [24]. Following inoculation, each well was sealed with a layer of sterile mineral oil to create a physical barrier against air and establish an oxygen-restricted environment (denoted as anaerobic condition) according to previous studies [25]. As indicated, metal compounds were supplemented into Mueller-Hinton broth (MHB; non-cation-adjusted, SanYao, Beijing, China) to achieve a final concentration of 100 μM according to a previous study [26]. Polyvinylpyrrolidone K30 (PVP, D&B Biological Science and Technology Co., Ltd., Shanghai, China; 10 mM in sterile water,) was also supplemented to promote the combined antibacterial activity in different sequence and heat treatment.

### 2.3. Bacterial Growth Curves and Time–Kill Curves

*E. coli* B2 was cultured in fresh LB broth at 37 °C with shaking at 220 rpm until the mid-logarithmic phase was reached. The bacterial suspension was then adjusted to 1 × 10^6^ CFU/mL using CAMHB. Subsequently, bacterial suspensions were combined 1:1 with either BAK (8 μg/mL), COL (1 μg/mL), or their combination in a 96-well plate. Growth curves were generated by measuring the OD600 at specified time intervals. Time–kill kinetics were assessed by quantifying viable bacteria at 0, 0.5, 1, 2, 4, and 8 h. At each time point, cultures were serially diluted 10-fold in sterile phosphate-buffered saline (PBS, pH 7.4), and 100 μL aliquots were spread onto Plate Count Agar (PCA). For spotting assays, 5 μL of each dilution was spotted onto Mueller-Hinton Agar (MHA, SanYao, Beijing, China). All plates were incubated overnight at 37 °C, and time–kill curves were plotted based on colony counts.

### 2.4. Checkerboard Assays

Synergistic interactions were quantified via checkerboard microdilution assays, with the Fractional Inhibitory Concentration Index (FICI) calculated as follows: FICI = FICI_a_ + FICI_b_ = MIC_ab_/MIC_a_ + MIC_ba_/MIC_b_. MIC_a_ is the MIC of compound A alone; MIC_ab_ is the MIC of compound A in combination with compound B; MIC_b_ is the MIC of compound B alone; MIC_ba_ is the MIC of compound B in combination with compound A. FICI values were interpreted as follows: ≤0.5, synergy; >2, antagonism. All assays were performed in triplicate unless specified otherwise.

### 2.5. Lipid Competitive Assays

Supplemental phospholipids (32 μg/mL each) were incorporated into MHB (non-cation-adjusted) as follows: phosphorylethanolamine (Pro-PE; Adamas, Shanghai, China, 10 mg/mL in sterile water), phosphatidylethanolamine (PE, Solarbio, Beijing, China; 10 mg/mL in ethanol), lysophosphatidylcholine (Lyso-PC, Rhawn, Shanghai, China; 10 mg/mL in sterile water), 18:1 cardiolipin disodium salt (CL, Macklin, Shanghai, China; 25 mg/mL in methanol), phosphatidylcholine (PC, Rhawn, Shanghai, China; 25 mg/mL in ethanol), L-α-phosphatidylglycerol (PG, Aladdin, Shanghai, China; 25 mg/mL in DMSO), and lecithin (Polyene-PC, Macklin, Shanghai, China; 10 mg/mL in DMSO).

### 2.6. Osmotic Stress Testing

Antimicrobial susceptibility under osmotic stress was assessed using modified MHB formulated with 17.5 g/L tryptone, 2.5 g/L beef extract, and graded NaCl concentrations: hypotonic (1.0 g/L), isotonic (5.0 g/L), or hypertonic (15.0 g/L). After autoclaving, the pH was adjusted to 7.4 using a pH meter.

### 2.7. ROS Accumulation and Lipid Peroxidation Assay

Intracellular reactive oxygen species (ROS) accumulation was measured using 2′,7′-dichlorodihydrofluorescein diacetate (DCFH-DA; 10 μM, excitation/emission wavelengths (Ex/Em) = 480/530 nm, Beyotime, Shanghai, China). Nitric oxide production was assessed using diaminofluorescein-2 diacetate (DAF-2DA; Ex/Em = 480/530 nm, Aladdin, Shanghai, China). Lipid peroxidation was detected with diphenyl-1-pyrenylphosphine (DPPP; 10 μM, Ex/Em = 351/380 nm, Rhawn, Shanghai, China), while neutral lipids were stained with BODIPY 493/503 (10 μM, Ex/Em = 473/513 nm, Bide, Shanghai, China), which readily crosses cell membranes.

### 2.8. ATP Quantification

ATP levels were measured using an enhanced ATP assay kit (Beyotime, Shanghai, China). *E. coli* B2 was treated with PBS (control), BAK (8 μg/mL), COL (1 μg/mL), or the combination. After 30 min incubation, samples were centrifuged (8000 rpm, 5 min, 25 °C) to collect supernatant for extracellular ATP measurement. Intracellular ATP was quantified from the lysed cell pellets. Luminescence was measured using a microplate reader.

### 2.9. PMF Measurement

The proton motive force (PMF), an essential electrochemical gradient for bacterial energy transduction, consists of both the electrical potential (Δψ) and the transmembrane pH gradient (ΔpH). Membrane potential (Δψ) was assessed with tetramethylrhodamine ethyl ester (TMRE, TargetMol, Boston, MA, USA; 10 μM, Ex/Em = 543/576 nm) in HEPES buffer (5 mM, pH 7.0) containing 20 mM glucose. The proton gradient (ΔpH) was measured using 2’,7’-bis-(2-carboxyethyl)-5(6)-carboxyfluorescein acetoxymethyl ester (BCECF-AM, 5 μM, Beyotime, Shanghai, China; Ex/Em = 490/535 nm), an intracellular pH-sensitive fluorescent probe.

### 2.10. EB Assay

Ethidium bromide (EB), a nucleic acid fluorescent dye, was employed to simultaneously assess alterations in bacterial membrane permeability and efflux pump activity [27]. Cells were incubated with EB (Macklin, Shanghai, China; 10 μM) in PBS containing BAK (8 μg/mL), COL (1 μg/mL), or the combination. Fluorescence was measured at Ex/Em = 530/590 nm using a microplate reader for 120 min.

### 2.11. Membrane Permeability

Bacterial membrane integrity was assessed by the uptake of 1-N-phenylnaphthylamine (NPN; Macklin, Shanghai, China) and propidium iodide (PI; Macklin, Shanghai, China) [28]. Fluorescence was measured at Ex/Em = 355/405 nm for NPN and 530/590 nm for PI.

### 2.12. Biofilm Inhibition Assay

Biofilm inhibition was quantified by crystal violet staining. Biofilms were formed by co-incubating late-logarithmic phase *E. coli* B2 cultures with various drug combinations in 96-well plates at 37 °C for 36 h. After removing planktonic cells, biofilms were gently washed twice with PBS, air-dried, stained with 200 μL 0.1% crystal violet for 20 min, and then washed three times with PBS. Following dissolution of the bound dyes in 33% (*v*/*v*) acetic acid, the absorbance was measured at a wavelength of 595 nm.

### 2.13. UV-Vis Spectroscopy

A working solution of BAK was prepared and combined with Fe^2+^/Fe^3+^ solutions (derived from 100 mM stock solutions of FeSO_4_/FeCl_3_ prepared in sterile water) to achieve final concentrations of 100 μM BAK and 100 μM of each iron ion. Serial dilutions of BAK were also mixed with PVP (7.8 μM). The interactions among BAK, Fe^2+^/Fe^3+^, and PVP were analyzed by UV-Vis spectroscopy, with scans performed from 230 to 450 nm at a resolution of 2 nm. All measurements were carried out in triplicate.

### 2.14. Intracellular Fe^2+^ Quantification

RhoNox-1 (MedChemExpress, Shanghai, China; 10 mM stock in DMSO), a cell-permeable probe that reacts irreversibly with ferrous iron (Fe^2+^) to generate fluorescent products, was used to quantify labile Fe^2+^ pools [29]. Fluorescence intensity (Ex/Em = 540/575 nm) was monitored at indicated time points using a microplate reader.

### 2.15. Hemolysis Assay

An 8% erythrocyte suspension was prepared in PBS using fresh sterile defibrinated sheep blood (Pingrui Technology, Zhengzhou, China). Test compounds, including two-fold serial dilutions of BAK alone (ranging from 8 to 128 μg/mL), BAK alone at 8 μg/mL, COL alone at 1 μg/mL, BAK-COL combinations, were mixed at a 1:1 ratio with the erythrocyte suspension in 1.5 mL tubes. After incubation at room temperature for 1 h, the supernatant was collected to measure the absorbance at 576 nm (OD576). PBS and 0.2% Triton X-100 served as the negative and positive controls, respectively. Hemolysis percentage was calculated as: Hemolysis (%) = (OD576 _sample_ − OD576 _PBS_)/(OD576 _0.2% Triton X-100_ − OD576 _PBS_) × 100%. All treatments were performed in triplicate.

### 2.16. In Vivo Therapeutic Evaluation

*Galleria mellonella* larvae (≈400 mg) were randomly divided into 4 groups (*n* = 10 per group). *E. coli* B2 was adjusted to 1 × 10^8^ CFU/mL in PBS, and 10 μL (1 × 10^6^ CFUs/larva) was injected into the last left proleg. After 1 h, treatments (10 μL) were administered into the last right proleg: (control, PBS, BAK (8 mg/kg), COL (1 mg/kg), or BAK + COL combination (8 + 1 mg/kg). Survival was monitored at 12 h intervals for 72 h.

### 2.17. Statistical Analysis

All statistical analyses were performed using GraphPad Prism 9.5.0. Biological replicates were included for all experiments. Unless otherwise specified. Data are presented as mean ± standard deviation (SD) unless otherwise specified. *p*-values were determined by one-way ANOVA, with *, *p* < 0.05 considered statistically significant.

## 3. Results

### 3.1. Synergistic Antibacterial Effects of BAK-COL Combination

**Figure 1:**
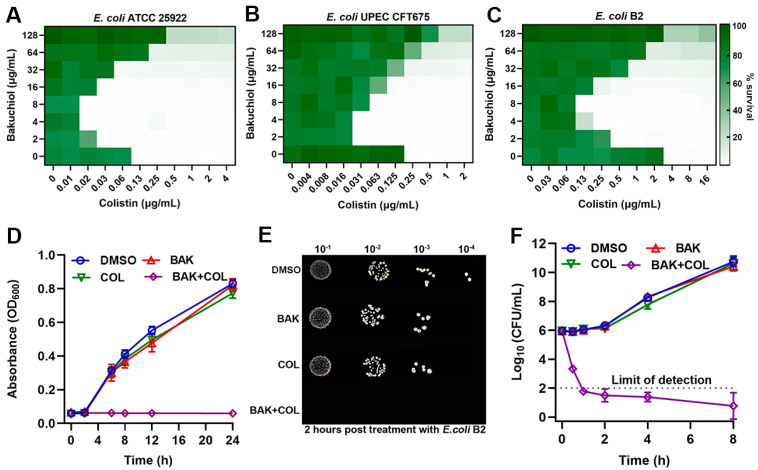
Bakuchiol (BAK) potentiates colistin (COL) activity. (**A**–**C**) Checkerboard broth microdilution assays evaluating BAK-COL combinations. (**D**) Growth kinetics of *E. coli* B2. (**E**) Spot assays of serial dilutions after 2 h treatment. (**F**) Time–kill curves of *E. coli* B2. In panels (**A**–**C**), darker green indicates higher bacterial density (OD600 values from two biological replicates). Concentrations in (**D**–**F**): BAK (8 μg/mL), COL (1 μg/mL), or combination.

To evaluate BAK therapeutic potential, checkerboard assays revealed that BAK alone (up to 128 μg/mL) showed no bactericidal activity (Figure 1A–C). However, BAK at low concentrations synergized with COL against *E. coli* ATCC 25922 (FICI = 0.16), clinical isolate *E. coli* CFT675 (FICI = 0.10).and MDR *E. coli* B2 (*mcr*-1-positive; FICI = 0.09), indicating an *mcr* independent mechanism. BAK decreased the MIC of COL against *E. coli* B2 from 4 μg/mL to 0.125 μg/mL. Growth curve analysis (Figure 1D), spot assays (Figure 1E) and time–kill curves (Figure 1F) confirmed the potent bactericidal synergy of the BAK-COL combination (8 + 1 μg/mL) against *E. coli* B2. However, at elevated concentrations exceeding 32 μg/mL, BAK exhibited attenuated or abolished synergistic enhancement of COL activity. The broad-spectrum applicability of this synergy was evidenced in multiple Gram-negative pathogens, including *Klebsiella pneumoniae*, *Pseudomonas aeruginosa*, *Salmonella enterica*, and *Acinetobacter baumannii* (Figure S1). The inverted U-shaped synergy profile likely stems from BAK self-assembly into hydrophobic aggregates, mediated by π-π stacking of phenolic rings.

### 3.2. BAK Induces Redox Imbalance and Metabolic Dysregulation

**Figure 2:**
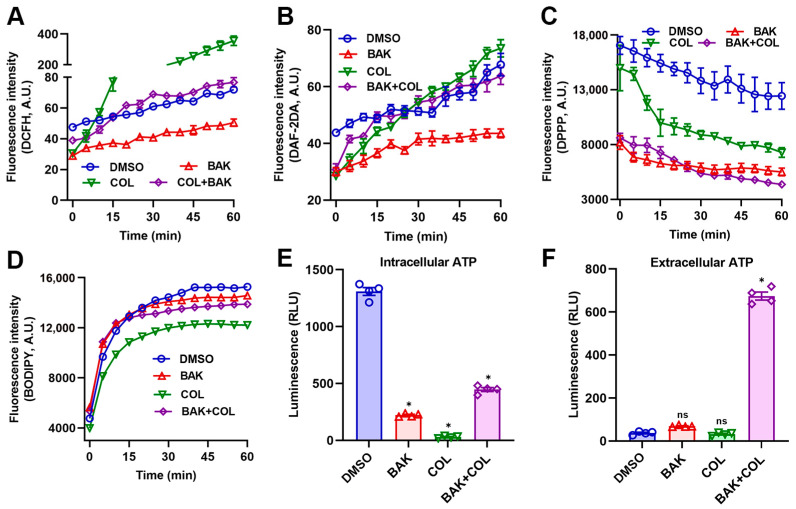
The BAK-COL combination induces metabolic dysregulation in *E*. *coli* B2. (**A**) DCFH-DA fluorescence (intracellular ROS). (**B**) DAF-2DA fluorescence (NO). (**C**) DPPP fluorescence (lipid peroxidation). (**D**) BODIPY 493/503 fluorescence (lipid droplets). (**E**) Intracellular ATP levels. (**F**) Extracellular ATP leakage. Treatments: Control (solvent vehicle), BAK (8 μg/mL), COL (1 μg/mL), or combination. Data represent mean ± SD. In (**E**,**F**), statistical differences were analyzed by ordinary one-way ANOVA Dunnett’s multiple comparisons to the DMSO groups. Significance levels were defined as: * *p* < 0.05.

Bacterial metabolic states intricately modulate antibacterial efficacy through multiple interconnected pathways [30]. COL at subinhibitory concentration (1 μg/mL) provoked significant reactive oxygen species (ROS) and nitric oxide (NO) generation in *E. coli* B2, as quantified by DCFH fluorescence and DAF-2D assays, respectively (Figure 2A,B). In stark contrast, BAK (8 μg/mL) suppressed both ROS and NO production, consistent with its documented antioxidant capacity [18]. Consequently, BAK counteracted COL-induced oxidative/nitrosative stress, a critical distinction from conventional adjuvants that amplify ROS for lethality [31]. In addition, DPPP and BODIPY 493/503 fluorescence staining further confirmed that the BAK-COL combination did not induce lipid peroxidation (Figure 2C,D). Critically, both COL and BAK monotherapies significantly inhibited intracellular ATP synthesis in *E. coli* B2 (Figure 2E). Concurrently, BAK-COL combination triggered massive ATP leakage (Figure 2F), indicating synergistic membrane poration may via dual-targeted damage to LPS and phospholipid bilayers.

### 3.3. BAK Disrupts Membrane Integrity and Function

**Figure 3:**
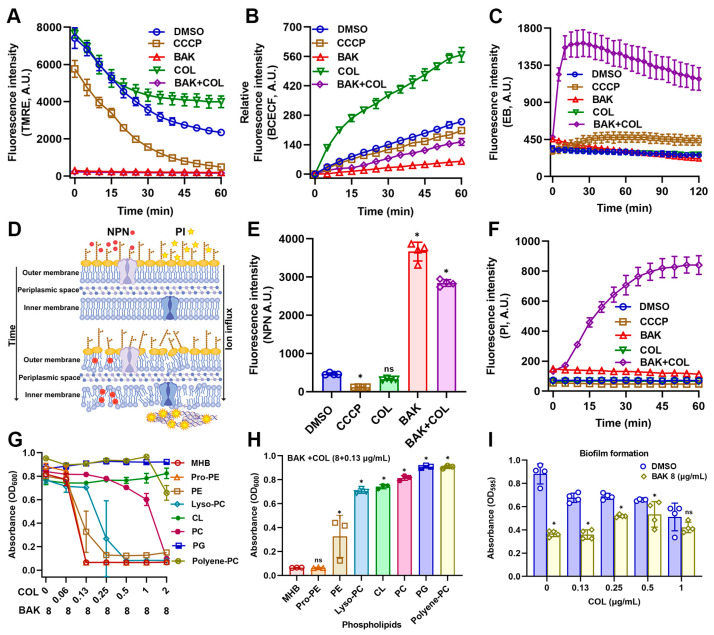
Effects of BAK and COL on membrane function and integrity in *E. coli* B2. (**A**) Membrane potential (Δψ) detected by TMRE staining. CCCP (25 μM; positive control for membrane potential inhibition). (**B**) Intracellular pH changes measured using BCECF-AM. (**C**) Membrane integrity and efflux pump activity monitored by EtBr (EB) uptake. (**D**) Schematic illustration of NPN/PI mechanisms. (**E**) Outer membrane permeability assessed by NPN uptake. (**F**) Membrane integrity evaluated by PI staining. (**G**) Competitive assays. Growth inhibition (OD600) of *E. coli* B2 treated with BAK (8 μg/mL) combined with varying COL concentrations in the presence of 32 μg/mL phospholipid components (Control, Pro-PE, PE, Lyso-PC, CL, PC, PG, and Polyene-PC) after 16 h incubation at 37 °C. (**H**) Growth inhibition (OD600) of *E. coli* B2 treated with COL (0.13 μg/mL) and BAK (8 μg/mL) combination supplemented with 32 μg/mL different phospholipids. (**I**) Biofilm formation assessed by crystal violet staining (OD595) under different COL concentrations (0–1 μg/mL) with or without BAK (8 μg/mL); Statistical differences were analyzed by two-way ANOVA. Treatment groups: BAK (8 μg/mL), COL (1 μg/mL), and combination treatment. Data represent mean ± SD. In (**E**,**F**,**H**,**I**), statistical differences were analyzed by ordinary one-way ANOVA Dunnett’s multiple comparisons to the DMSO groups. Significance levels were defined as: * *p* < 0.05.

Given the observed ATP leakage signifying the membrane integrity loss, we next investigated whether BAK-COL synergy directly disrupts the proton motive force (PMF), the electrochemical gradient essential for bacterial energy transduction [32]. PMF, composed of electrical potential (Δψ) and the transmembrane pH gradient (ΔpH), were assessed by TMRE and BCECF-AM, respectively. As shown in Figure 3A, both BAK-alone and BAK-COL combination treatments induced membrane potential dissipation. In addition, BAK suppressed pH elevation, impairing proton gradient maintenance (Figure 3B). The rapid and profound PMF dissipation suggested catastrophic membrane dysfunction extending beyond bioenergetic collapse [33]. To directly probe structural membrane integrity, we quantified membrane permeability using EtBr (EB), 1-N-phenylnaphthylamine (NPN), and PI uptake assays. As expected, the BAK-COL combination treatment led to a rapid increase in fluorescence, indicating significant disruption of membrane integrity (Figure 3C–F).

To investigate membrane-targeting mechanisms, *E. coli* B2 cultures were supplemented with various phospholipids (PE, Pro-PE, Lyso-PC, CL, PC, PG, or Polyene-PC at 32 μg/mL) prior to BAK-COL treatment (Figure 3G). Critically, supplementation with exogenous phospholipids, specifically CL, PC, and PG, significantly attenuated the antibacterial efficacy of the BAK-COL combination. (Figure 3H). This competitive inhibition provides direct evidence that the BAK-COL synergy is primarily associated with membrane phospholipids. Since these phospholipids organize the hydrophobic scaffold of Gram-negative biofilms, providing structural cohesion and limiting antibiotic penetration [34], we hypothesized superior biofilm inhibition by BAK-COL versus planktonic cells. As shown in Figure 3I, COL inhibited biofilm formation in a dose-dependent manner, while BAK (8 μg/mL) alone also significantly reduced biofilm formation. Collectively, these results establish BAK as a novel membrane-targeting adjuvant enhancing COL activity by specifically exploiting lipid vulnerabilities in multidrug-resistant Gram-negative pathogens.

### 3.4. BAK Disrupts Iron Homeostasis

**Figure 4:**
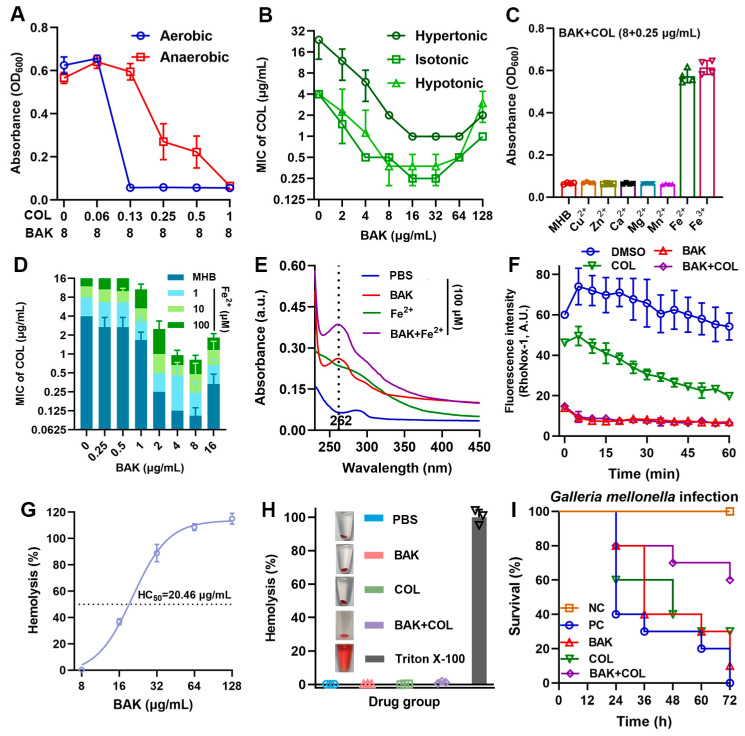
BAK disrupts iron homeostasis. (**A**) BAK (8 μg/mL) combined with varying COL concentrations against *E. coli* B2 under aerobic and anaerobic conditions (*n* = 3). (**B**) Antibacterial activity under different osmotic pressures (hypotonic, isotonic, and hypertonic MHB; *n* = 2). (**C**) Growth inhibition (OD600) of *E. coli* B2 treated with BAK (8 μg/mL) + COL (0.25 μg/mL) supplemented with 100 μM divalent cations (Cu^2+^, Zn^2+^, Ca^2+^, Mn^2+^, Mg^2+^, Fe^2+^, or Fe^3+^) after 18 h incubation (*n* = 4). (**D**) Dose-dependent effects of Fe^2+^ on BAK-COL activity (*n* = 3). (**E**) UV-Vis absorption spectra of BAK-Fe^2+^ interactions in PBS (*n* = 3). (**F**) Intracellular free Fe^2+^ levels (RhoNox-1 fluorescence) in *E. coli* B2 treated with BAK (8 μg/mL), COL (1 μg/mL), or their combination (*n* = 4). Data represent mean ± SD. (**G**) Hemolysis percentage of erythrocytes exposed to increasing BAK concentrations (8–128 μg/mL), with HC50 determination (*n* = 3). (**H**) Hemolytic percentage of erythrocytes treated with positive control (0.2% Triton X-100), PBS, BAK (8 μg/mL), COL (1 μg/mL), or their combination (*n* = 3). (**I**) Survival rates of *E. coli* B2-infected *Galleria mellonella* larvae (1 × 10^6^ CFUs) treated with BAK (8 mg/kg), COL (1 mg/kg), or their combination (*n* = 10), negative control (NC), positive control (PC).

Since PMF generation requires terminal electron acceptors unavailable in anaerobiosis, we hypothesized strict oxygen dependence for synergistic killing. As expected, the synergistic antibacterial activity of BAK-COL significantly attenuated under anaerobic conditions (Figure 4A). Moreover, hyperosmotic stress compromised the antibacterial activity of COL but preserved the synergistic potentiation of BAK, reducing the MICs of COL by 8-fold against *E. coli* B2 (Figure 4B). Notably, among various divalent cations tested, only iron ions (Fe^2+^/Fe^3+^) at 100 μM significantly reduced the antibacterial activity of the combination (Figure 4C). Iron is a critical component of bacterial respiratory Fe-S clusters and heme groups, explaining the oxygen-dependent synergy [35,36]. Specifically, to clarify the role of iron ions in the enhanced efficacy of BAK in combination with COL, we determined the binding between BAK and iron ions using spectral scanning. The results revealed that the absorbance of BAK at λ_max_ = 262 nm increased upon of Fe^2+^ or Fe^3+^ addition, indicating BAK–Fe complex formation (Figure 4E, Supplementary Figure S2). Consistent with prior studies on iron-targeting adjuvants [15,37,38], BAK also enhances COL efficacy by disrupting bacterial iron homeostasis. RhoNox-1 fluorescence confirmed that BAK alone and in combination with COL significantly reduced intracellular free Fe^2+^ levels (Figure 4F). Hemolysis assays demonstrated dose-dependent erythrocyte lysis with an HC50 of 20.46 μg/mL for BAK (Figure 4G). Notably, neither BAK (8 μg/mL), COL (1 μg/mL), nor their combination exhibited hemolytic activity at therapeutic concentrations (Figure 4H). In the *E. coli* B2 infected *Galleria mellonella* model (Figure 4I), the BAK-COL combination significantly enhanced 72 h larval survival compared to monotherapies. Collectively, these data demonstrate that the adjuvant activity of BAK is oxygen-dependent but osmotolerant, with iron chelation serving as the non-redundant mechanism for COL synergy.

### 3.5. PVP Promotes the Synergistic Activity of BAK-COL Combination

**Figure 5:**
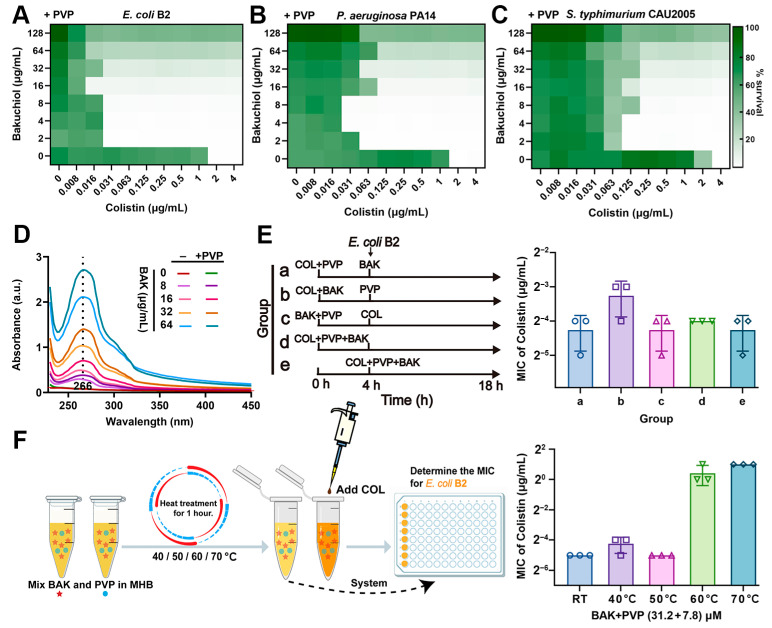
Influence of PVP on the antibacterial efficacy of BAK-COL combination. (**A**–**C**) Inhibitory effects against *E. coli* B2, *P. aeruginosa* PA14, and *S. typhimurium* CAU2005 by BAK-COL combinations in MHB containing 7.8 μM PVP. Darker green indicates greater bacterial growth. (**D**) UV-Vis absorption spectra (230–450 nm) of BAK with or without 7.8 μM PVP. (**E**) Experimental workflow for sequential treatment with BAK (8 μg/mL), COL (1 μg/mL), and PVP (7.8 μM), and its impact on MICs of COL against *E. coli* B2. (**F**) Thermal stability assessment: workflow for heat-treated BAK-PVP (31.2 + 7.8 μM) and subsequent MIC determination with COL against *E. coli* B2.

The therapeutic potential of BAK is limited by its poor aqueous solubility at high concentrations. To address these limitations, we incorporated polyvinylpyrrolidone (PVP), an FDA-approved hydrophilic polymer that it demonstrates versatile applications in wound healing dressings, nano-delivery systems for enhanced bioavailability, and biofilm formation inhibition [39]. Notably, PVP supplementation significantly potentiated BAK-COL activity against AMR Gram-negative pathogens (*E. coli* B2, *P. aeruginosa* PA14, and *S. typhimurium* CAU2005) while extending the therapeutic window to 128 μg/mL, overcoming concentration-dependent limitations (Figure 5A–C and Figure S2B,C). UV-Vis spectral analysis confirmed the direct molecular interactions between BAK and PVP (Figure 5D). The sequence-independent (Figure 5E) and thermostable (<60 °C) nature (Figure 5F) of BAK-COL synergy eliminates administration timing constraints and allows lyophilized storage, critical advantages for resource-limited settings. Collectively, PVP emerges as a formulation essential for BAK-COL translation, preventing concentration-dependent attenuation.

## 4. Discussion

The escalating crisis of antimicrobial resistance, particularly among Gram-negative pathogens, represents a critical challenge to public health. It is essential to develop innovative strategies to extend the efficacy of last-resort antibiotics such as colistin. In this study, we identify the natural meroterpenoid bakuchiol—recognized for its notable antioxidant properties—as a highly effective adjuvant that synergistically enhances colistin activity against a panel of MDR Gram-negative clinical isolates.

**Figure 6:**
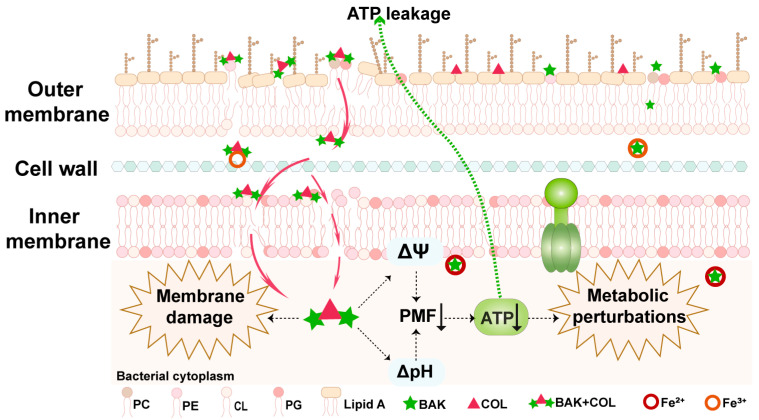
A schematic model illustrating the mechanism of BAK in synergy with COL. The BAK-COL combination interacts with both outer membrane (OM) Lipid A of LPS and phospholipids (PC, PE, CL, PG) in OM, disrupting membrane integrity and causing massive ATP leakage. This triggers membrane depolarization, ATP synthesis inhibition, and bacterial iron homeostasis disruption, collectively inducing membrane dysfunction and ultimately leading to metabolic collapse.

Notably, the synergy observed is not merely additive but stems from a compelling dual mechanism that disrupts two fundamental pillars of bacterial physiology: iron homeostasis and membrane integrity. While several previous studies have reported membrane-targeting activities of natural compounds [13,15], the capacity of BAK to concurrently chelate intracellular iron represents a distinct and underappreciated mode of action. BAK disrupts bacterial iron homeostasis leading to iron depletion, a critical factor for bacterial survival and virulence. This iron depletion likely induces a state of iron starvation, disrupting iron-dependent metabolic pathways and potentially generating iron-mediated oxidative stress [40]. Subsequently, BAK uniquely suppresses rather than accumulates ROS levels. When co-administered with COL, BAK also significantly reduces COL-induced ROS generation, consistent with BAK’s antioxidant properties. It is plausible that the inherent antioxidant nature of BAK contributes to its own stability and may modulate the redox environment within the bacterial cell, further exacerbating metabolic dysfunction. The consequential collapse of the proton motive force, essential for ATP production and nutrient transport, appears to be a terminal event resulting from this multipronged assault [32]. Concurrently, BAK interacts directly with membrane phospholipids, compromising membrane integrity (Figure 6). The critical next steps involve resolving temporal iron-membrane interactions using in situ cryo-electron tomography to visualize pore formation in Fe^2+^ depleted bacteria. Thus, the ROS-independent bactericidal mechanism of BAK distinctly differs from that of conventional antibiotics [40].

Given its ability to compromise membrane integrity and disrupt iron homeostasis, it is plausible that BAK also interferes with the assembly and stability of the outer membrane in Gram-negative bacteria. Specifically, BAK may perturb the transport or assembly of lipopolysaccharide (LPS), a critical component for outer membrane impermeability. By interacting with membrane phospholipids and potentially with proteins involved in the LPS transport machinery (such as the Lpt system) [41], BAK could induce mislocalization or defective insertion of LPS, thereby weakening membrane integrity and enhancing permeability to COL and other antibiotics.

In addition, the BAK–COL combination showed no detectable cytotoxicity and exhibited significant in vivo efficacy in a *Galleria mellonella* infection model, supporting its potential therapeutic utility. Here, we addressed a common hurdle in the therapeutic application of lipophilic natural compounds—poor aqueous solubility—by employing PVP as a dispersant. PVP has been demonstrated to enhance the solubility of hydrophobic compounds via hydrogen bonding and hydrophobic interactions, while also improving drug stability through the protection of phenolic hydroxyl groups [42,43]. The successful stabilization of BAK’s synergistic activity across a wide concentration range through PVP formulation provides a practical pharmaceutical strategy to enhance its bioavailability and efficacy, moving the combination closer to potential clinical application. Formulation innovations should prioritize nanoparticle engineering [42,43] for targeted biofilm penetration in device-associated infections.

However, the findings of this study must be interpreted within its limitations. The exact contribution of its antioxidant property versus its pro-oxidant potential in the bacterial context warrants deeper investigation. While we established a clear role for iron dysregulation, the precise molecular targets of BAK, especially concerning its interaction with bacterial iron acquisition, storage, and regulatory systems, remain to be fully elucidated. Transcriptomic and proteomic analyses may reveal global changes in gene expression upon BAK exposure. Finally, the in vivo infection model (*Galleria mellonella*) only provides valuable insights into efficacy and toxicity; but does not fully recapitulate the complex immune responses and pharmacokinetic profiles of mammalian systems. Despite the current limitations, we provide a strong foundation for further exploration of natural antioxidants as multifunctional agents in the relentless battle against multidrug-resistant infections.

## 5. Conclusions

This study demonstrates that the BAK-COL combination exhibits potent synergistic antimicrobial activity against multidrug-resistant Gram-negative strains. BAK disrupts iron homeostasis and simultaneously compromises membrane integrity through phospholipid binding. Furthermore, formulation with water-insoluble BAK and PVP carrier not only enhanced the antibacterial efficacy but also expanded the effective concentration range of BAK-COL. These findings establish a novel therapeutic strategy of antioxidants against MDR bacterial infections.

## Data Availability

The data that support the findings of this study are available from the corresponding authors upon reasonable request.

## Supplementary Materials

The following supporting information can be downloaded at: https://www.mdpi.com/article/10.3390/antiox14101178/s1, Figure S1: Checkerboard assays of BAK-COL against Gram-negative bacterial; Figure S2: Effects of Fe^3+^ on BAK-COL activity.

## Abbreviations

BAK: bakuchiol; COL, colistin; AMR, antimicrobial-resistant; MDR, multidrug-resistant; WHO, World Health Organization; LPS, lipopolysaccharides; PVP, polyvinylpyrrolidone K30; LB, Luria–Bertani; DMSO, Dimethyl sulfoxide; MIC, minimal inhibit concentration; CLSI, Clinical and Laboratory Standards Institute; FICI, Fractional Inhibitory Concentration Index; CAMHB, cation-adjusted Mueller-Hinton broth; MHB, Mueller-Hinton broth; CFU, colony-forming unit; Pro-PE, phosphorylethanolamine; PE, phosphatidylethanolamine; Lyso-PC, lysophosphatidylcholine; CL, 18:1 cardiolipin disodium salt; PC, phosphatidylcholine; PG, L-α-phosphatidylglycerol; Polyene-PC, lecithin; ROS, reactive oxygen species; NO, nitric oxide; PBS, phosphate-buffered saline; MHA, Mueller-Hinton; PCA, plate count agar; ATP, adenosine triphosphate; Ex/Em, excitation/emission wavelengths; DCFH-DA, 2′,7′-dichlorodihydrofluorescein diacetate; DAF-2DA, diaminofluorescein-2 diacetate; DPPP, diphenyl-1-pyrenylphosphine; PMF, proton motive force; ΔpH, pH gradient; Δψ, electrical potential; BCECF-AM, 2’,7’-bis-(2-carboxyethyl)-5(6)-carboxyfluorescein acetoxymethyl ester; TMRE, tetramethylrhodamine ethyl ester; CCCP, Carbonyl cyanide 3-chlorophenylhydrazone; EB, Ethidium bromide; NPN, 1-N-phenylnaphthylamine; PI, Propidium iodide; SD, Standard deviation; ANOVA, Analysis of Variance; FDA, Food and Drug Administration.
