# Supplementary material for: Bakuchiol, a Natural Antioxidant, Synergizes with Colistin Against Multidrug-Resistant Gram-Negative Bacteria by Disrupting Iron Homeostasis and Membrane Integrity
Source: Antioxidants (Basel). 2025 Sep 26;14(10):1178. doi: 10.3390/antiox14101178 (PMC12561805; doi:10.3390/antiox14101178)
Supplement: Supplementary file 1 [file antioxidants-14-01178-s001.zip › antioxidants-3871938-supplementary.pdf]

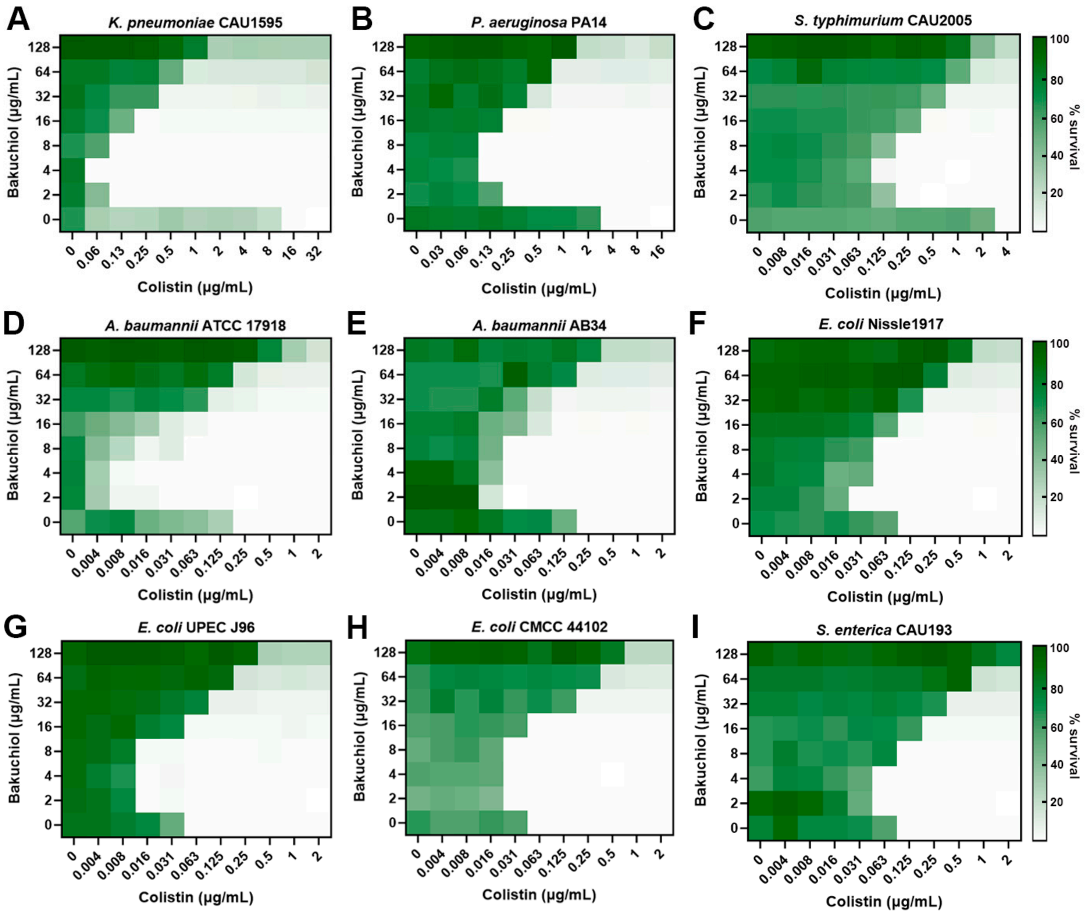

Figure S1. Checkerboard assays of BAK-COL against Gram-negative bacterial. Darker green indicates higher bacterial density (OD600 values from two biological replicates.

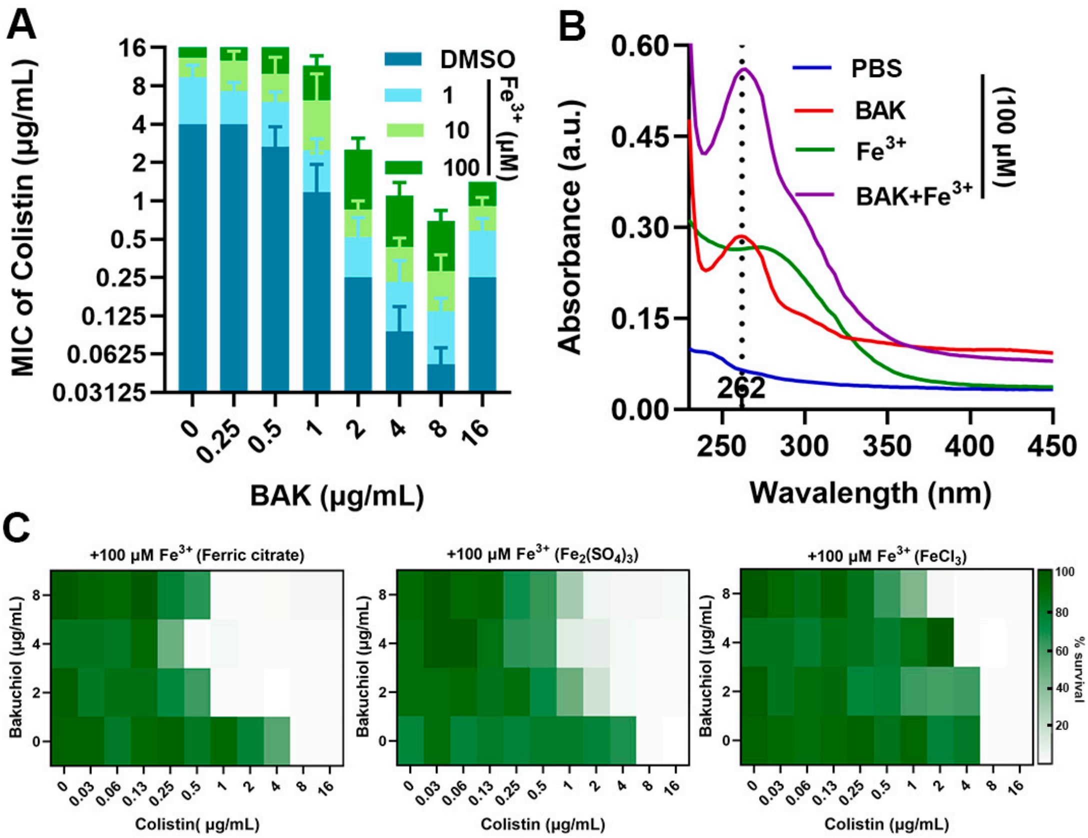

Figure S2. Effects of Fe^{3+} on BAK-COL activity. (A) Dose-dependent effects of Fe^{3+} on BAK-COL activity. Data represent mean ± SD. (B) UV-Vis absorption spectra of BAK-Fe^{3+} interactions in PBS. (C) Checkerboard assay heatmap depicting the antibacterial effects of BAK-COL combinations in MHB supplemented with different iron sources (100 μM).
